# Validation of oral fluid samples to monitor serological changes to *Plasmodium falciparum*: An observational study in southern Zambia

**DOI:** 10.1186/1475-2875-10-162

**Published:** 2011-06-10

**Authors:** Alexis P Chidi, Sandra Chishimba, Tamaki Kobayashi, Harry Hamapumbu, Sungano Mharakurwa, Philip E Thuma, William J Moss

**Affiliations:** 1 Johns Hopkins Bloomberg School of Public Health, Baltimore, MD, USA; 2 Macha Research Trust, Choma, Zambia

## Abstract

**Background:**

In formerly endemic areas where malaria transmission has declined, levels of population immunity to *Plasmodium falciparum *provide information on continued malaria transmission and potentially susceptible populations. Traditional techniques for measuring serological responses to *P. falciparum *antigens use plasma or dried blood spots (DBS). These invasive procedures pose a biohazard and may be unacceptable to communities if performed frequently. The use of oral fluid (OF) samples to detect antibodies to *P. falciparum *antigens may be a more acceptable strategy to monitor changes in population immunity.

**Methods:**

An enzyme immunoassay was optimized to detect antibodies to whole, asexual stage *P. falciparum *antigens. Optical density (OD) values from paired DBS and OF samples collected as part of a community-based survey of malaria parasitaemia were compared.

**Results:**

Oral fluid and dried blood spot samples were collected from 53 participants in Southern Province, Zambia. Their ages ranged from 1 to 80 years and 45% were female. A statistically significant correlation (r = 0.79; P < 0.01) was observed between OD values from OF and DBS samples. The OF assay identified all DBS-confirmed positive and negative samples, resulting in 100% sensitivity and specificity.

**Conclusions:**

Oral fluid is a valid alternative specimen for monitoring changes in antibodies to *P. falciparum *antigens. As OF collection is often more acceptable to communities, poses less of a biohazard than blood samples and can be performed by community volunteers, serological surveys using OF samples provide a strategy for monitoring population immunity in regions of declining malaria transmission.

## Background

In formerly malaria endemic areas where transmission has declined, changing levels of population immunity to *Plasmodium falciparum *provide information on foci of malaria transmission and potentially susceptible populations [1]. Serological studies can reveal more than the point prevalence of malaria and reflect secular trends in the level of exposure, and are thus less affected by seasonal variations in transmission [1,2]. Traditional serological techniques using plasma samples or dried blood spots (DBS) to detect antibodies to *P. falciparum *antigens pose a biohazard and may be unacceptable to communities if performed frequently [3].

Oral fluid (OF) refers to crevicular fluid, the transudate from the crevice between the gum margin and teeth, collected from the mouth using an absorptive device [4]. Crevicular fluid contains the highest concentration of immunoglobulins outside the blood, although these levels are approximately 1/1,000 of the concentration found in plasma. OF collection is less invasive and may encourage participation among groups such as children, pregnant women and the elderly, who may be averse to phlebotomy [5]. OF collection allows for sampling to be conducted by lower level health care workers or community volunteers, and poses a less significant biohazard than DBS collection or phlebotomy. OF has been used routinely to detect antibodies to human immunodeficiency [6], measles [7] and rubella [8] viruses among other pathogens.

The use of OF specimens to monitor changes in antibody levels to *P. falciparum *antigens may be a more acceptable strategy to monitor changes in population immunity, particularly in regions of declining malaria transmission. However, no published evidence exists on the use of OF to detect antibodies to *P. falciparum*. The use of OF samples to measure antibody levels to *P. falciparum *antigens was validated in a region of declining malaria transmission in southern Zambia.

## Methods

The study was conducted in the catchment area of Macha Hospital in Southern Province, Zambia. Macha Hospital is approximately 70 km from the nearest town of Choma and the catchment area is populated by traditional villagers living in small, scattered homesteads. *Anopheles arabiensis *is the primary vector responsible for malaria transmission [9], which peaks during the rainy season from December through April. Over the past decade the Southern Province of Zambia has experienced a substantial decline in the burden of malaria [10].

Satellite images were used to construct a sampling frame for the random selection of households. Permission from the chief and head of household were obtained prior to the study visits. Field workers obtained individual informed consent and a questionnaire was administered to each study participant to collect demographic information as well as information on prior malaria infections and treatment history. Blood samples were collected by finger prick and stored as DBS on filter paper (Whatman, Protein Saver card 903). The cards were dried overnight and stored individually with desiccant in sealed plastic bags. OF samples were obtained from the same participants using Aware Messenger oral-specimen collection devices (Calypte Biomedical Corporation, Portland, OR, USA). Participants' gums were swabbed for one minute according to the manufacturer's instructions, and samples were transferred to a collection tube with the manufacturer's transport buffer. Samples were stored at room temperature. The study was approved the Johns Hopkins Bloomberg School of Public Health Institutional Review Board and the University of Zambia Research Ethics Committee.

An enzyme immunoassay (EIA) was used to detect antibodies to whole, asexual stage *P. falciparum *antigens with known positive and negative control samples (Table 1). Sera were eluted from DBS using 5% skim milk in phosphate buffered saline with 0.05% Tween 20 (PBST). OF samples were centrifuged for 5 minutes at 14,000 rpm and 75 μL of the supernatant was added to 25 μL of 10% skim milk with PBST in each well before the plate was incubated for 15 hours at 4°C. Whole *P. falciparum *asexual stage antigens were coated on a flat-bottomed 96 well plate (Thermo, Immulon 2HB) overnight at 4°C. The plate was blocked using 5% skim milk with phosphate buffered saline for one hour at 37°C. Serum and OF samples were plated in triplicate and incubated for one hour at 37°C, and peroxidase-labeled goat anti-human IgG was added to the plate for one hour at 37°C. ABTS was added to the plate for 15 minutes at room temperature before the optical density (OD) was read at 405 nm.

**Table 1:** Optimization conditions for oral fluid enzyme immunoassay

Variable	Conditions Tested
Volume of solution in well	100 μL or 200 μL
Incubation time	1, 2, 6, 8, or 15 hours
Incubation temperature	4°C or 37°C
Dilution	OF alone, 1:2, 1:4 with PBST, 5% skim milk PBST
Proportion of sample on plate	75 μL OF + 25 μL diluent or 50 μL OF + 50 μL diluent
Centrifugation (5 min at 14000 rpm)	None, supernatant, resuspended pellet

OF = oral fluid

PBST = phosphate buffered saline with 0.05% Tween 20

To classify samples as seropositive or seronegative, test results were compared to a threshold OD level established as three standard deviations above the OD observed from filter paper spotted with serum from individuals who reported never having been exposed to malaria. OD values from paired DBS and OF samples were compared using Stata version 11.0 and the correlation coefficient was computed.

## Results

**Figure 1:**
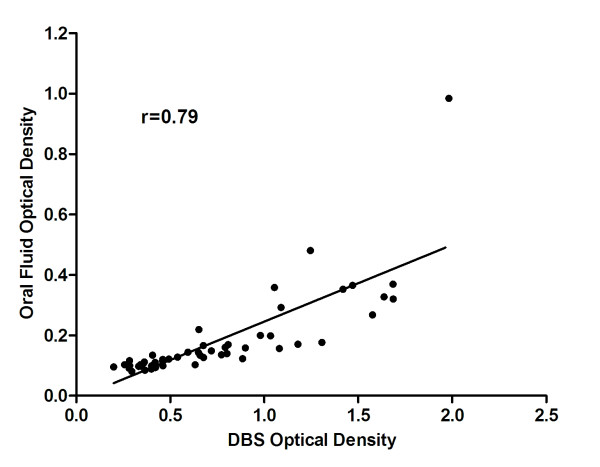
**Scatter plot showing correlation between optical density values obtained using oral fluid and dried blood spot samples**.

**Figure 2:**
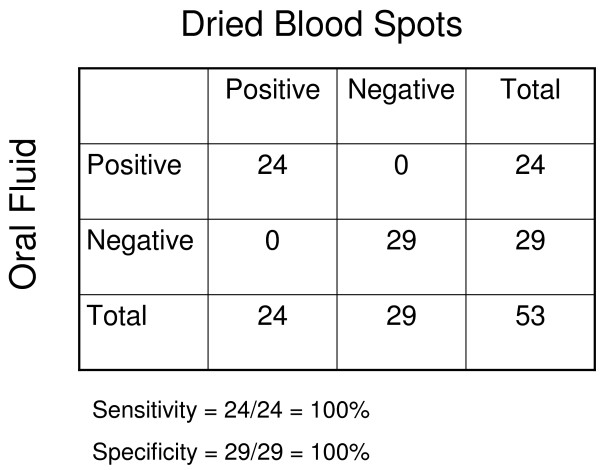
**Comparison of enzyme immunoassay results using oral fluid and dried blood spots**.

Oral fluid and dried blood spot samples were collected from 53 participants in Southern Province, Zambia. Their ages ranged from 1 to 80 years and 45% were female. The median age was 13.5 years and 17% were younger than five years of age. A statistically significant correlation (r = 0.79, r^2 ^= 0.63, P < 0.01) was observed between OD values obtained from OF and DBS samples (Figure 1). When the single outlier was excluded from analysis, the correlation improved (r = 0.84, r^2 ^= 0.71). However, OD values obtained from OF samples were consistently lower than those obtained from DBS. The OF assay identified all DBS-confirmed positive and negative samples, resulting in 100% sensitivity and specificity (Figure 2).

## Discussion

These results suggest that OF is a valid alternative specimen for monitoring changes in antibodies to *P. falciparum*. OF samples are likely to be more acceptable for community surveillance, particularly in areas of declining malaria transmission and when repeated measures are desired. As OF collection poses less of a biohazard than blood samples, and can be performed by unskilled volunteer health workers as part of task shifting [11], the use of OF for antibody determination allows for frequent testing in resource-limited settings.

OF samples have been used for serological surveillance of a variety of infections [12], and have been used to detect *P. falciparum *DNA by polymerase chain reaction [13-15] and histidine-rich protein 2 by EIA [16]. These results suggest that OF samples also can be used to monitor changes in population immunity as a component of malaria surveillance programs. The major limitation of OF samples is the difficulty in assessing whether an adequate sample was collected. Detection of total IgG is one strategy to identify improperly collected samples [7].

## Conclusions

OF sampling provides an alternative method of conducting surveillance for malaria population immunity and transmission. As malaria control programmes successfully reduce transmission, surveillance will be increasingly important to guide control strategies. The use of OF samples could make repeated surveys acceptable and feasible in low-resource settings.

## Abbreviations

ABTS: 2,2'-azino-bis(3-ethylbenzthiazoline-6-sulphonic acid); DBS: dried blood spots; EIA: enzyme immunoassay; OD: optical density; OF: oral fluid; PBST: phosphate buffered saline with 0.05% Tween 20

## Competing interests

The authors declare that they have no competing interests.

## Authors' contributions

APC performed the enzyme immunoassays and drafted the manuscript. SC performed and supervised the enzyme immunoassays. TK designed the experiments, supervised the enzyme immunoassays and participated in the preparation of the manuscript. HH participated in the design and coordination of the data and sample collection. SM participated in the design and coordination of the study. PET participated in the design and coordination of the study. WJM conceived of the study, participated in its design and coordination, and drafted the manuscript. All authors read and approved the final manuscript.
